# Widespread Frequent Methane Emissions From the Oil and Gas Industry in the Permian Basin

**DOI:** 10.1029/2022JD037479

**Published:** 2023-01-31

**Authors:** J. P. Veefkind, R. Serrano‐Calvo, J. de Gouw, B. Dix, O. Schneising, M. Buchwitz, J. Barré, R. J. van der A, M. Liu, P. F. Levelt

**Affiliations:** ^1^ Royal Netherlands Meteorological Institute KNMI De Bilt The Netherlands; ^2^ Department of Geoscience and Remote Sensing Delft University of Technology Delft The Netherlands; ^3^ Cooperative Institute for Research in Environmental Sciences University of Colorado Boulder Boulder CO USA; ^4^ Department of Chemistry University of Colorado Boulder Boulder CO USA; ^5^ Institute of Environmental Physics (IUP) University of Bremen FB1 Bremen Germany; ^6^ University Cooperation for Atmospheric Research Boulder CO USA; ^7^ National Center for Atmospheric Research Boulder CO USA

**Keywords:** methane emission, Tropomi satellite, Permian basin, oil and gas industry

## Abstract

Emissions of methane (CH_4_) in the Permian basin (USA) have been derived for 2019 and 2020 from satellite observations of the Tropospheric Monitoring Instrument (TROPOMI) using the divergence method, in combination with a data driven method to estimate the background column densities. The resulting CH_4_ emission data, which have been verified using model data with known emissions, have a spatial resolution of approximately 10 km. The CH_4_ emissions show moderate spatial correlation with the locations of oil and gas production and drilling activities in the Permian basin, as well as with emissions of nitrogen oxides (NO_x_). Analysis of the emission maps and time series indicates that a significant fraction of methane emissions in the Permian basin is from frequent widespread emissions sources, rather than from a few infrequent very large unplanned releases, which is important considering possible CH_4_ emission mitigation strategies. In addition to providing spatially resolved emissions, the divergence method also provides the total emissions of the Permian basin and its main sub‐basins. The total CH_4_ emission of the Permian is estimated as 3.0 ± 0.7 Tg yr^−1^ for 2019, which agrees with other independent estimates based on TROPOMI data. For the Delaware sub‐basin, it is estimated as 1.4 ± 0.3 Tg yr^−1^ for 2019, and for the Midland sub‐basin 1.2 ± 0.3 Tg yr^−1^. In 2020 the emissions are 9% lower compared to 2019 in the entire Permian basin, and respectively 19% and 27% for the Delaware and Midland sub‐basins.

## Key Points

- Methane emissions from the Permian basin in the USA can be derived from satellite data with a spatial resolution of approximately 10 km
- The derived emissions are spatially moderately correlated with satellite‐derived NO_x_ emissions and oil and gas industry activities
- A significant fraction of methane emissions in the Permian basin is from frequent widespread emissions sources related to the oil and gas industry

## 1 Introduction

Methane (CH_4_) is the second most important anthropogenic greenhouse gas after carbon dioxide (CO_2_). As the atmospheric lifetime of CH_4_ is relatively short at 9.1 ± 0.9 years (Masson‐Delmotte et al., 2021) and the global warming potential large, a reduction in CH_4_ emissions would lower the combined radiative forcing from greenhouse gases on a timescale of years making it a relatively efficient option to mitigate climate change. For this reason, the Global Methane Pledge was initiated at the UN Climate Change Conference (COP26) in November 2021 (European Commission, United States of America, 2021), which aims at reducing CH_4_ emissions by 30% by 2030.

A significant fraction of global methane emissions comes from the oil and gas (O&G) industry (IEA, 2021). CH_4_ is emitted during the construction of new wells, when operating, during storage and transportation of oil and gas, and when wells are abandoned. Some of the emissions are intended releases, for example, from venting, while others are unintentional and caused by malfunctioning equipment or by accidents. The CH_4_ emissions of the oil and gas supply chain are estimated as 13 ± 2 Tg yr^−1^(Alvarez et al., 2018) in the USA in 2015, contributing more than one‐third of the total anthropogenic CH_4_ emissions in North America (Maasakkers et al., 2021). To be able to effectively reduce CH_4_ emissions from the O&G industry, the largest contributions must be known, as well as which ones can be mitigated with the least effort. In the recent literature there has been a strong focus on the detection of large emissions (aka super‐emitters) in O&G production regions using satellite data (Cusworth et al., 2021; Irakulis‐Loitxate et al., 2021; Lauvaux et al., 2022), for example, in Turkmenistan (Irakulis‐Loitxate et al., 2022) and Algeria (Varon et al., 2021). In this paper, we focus on the Permian basin, which is the region with the largest oil production (approximately 5.5 million barrels per day in 2022) and the second in gas production (approximately 600 million m^3^ per day in 2022) in the USA (https://www.eia.gov/petroleum/drilling/, last accessed 25‐10‐2022). The Permian basin is located in Texas and New Mexico and covers an area of approximately 160,000 km^2^. The exploitation of the Permian basin is mostly done using non‐conventional technologies, including hydraulic fracturing and horizontal drilling. The area is characterized by thousands of production facilities and new ones are continuously developed, while others are abandoned when no longer productive. Observations from the ground (Robertson et al., 2020), from aircraft and satellites have shown significant emission of CH_4_ but also other gases like nitrogen oxides (NO_x_) (e.g., De Gouw et al., 2020). Although the NO_x_ emissions come from different sources, for example, from the use of heavy machinery and power generators, they are expected to come from the same sites where the CH_4_ is emitted (Warneke et al., 2014). Because of the shorter lifetime, estimating NO_x_ emissions from spaceborne observations is simpler and may be used as a proxy for CH_4_ emissions (Roberts et al., 2022), provided that there is sufficient correlation between these emissions.

For reducing the CH_4_ emissions, a key question is if these emissions are dominated by a few infrequent large point sources or caused by frequent widespread smaller emissions. This is important because large emissions from a few facilities will be easier to reduce than small emissions from many facilities (Mayfield et al., 2017). For CH_4_ emissions in the Permian basin, we can think of the following scenarios:

1. widespread frequent emissions from thousands of facilities;
2. frequent emissions from a limited number of facilities;
3. infrequent emissions from a limited number of facilities.

Depending on which scenario dominates the total emission in the Permian basin, different mitigation measures may be required, where the scenarios 2 and 3 are expected to be mitigated with less effort compared to scenario 1. To address the question which scenario is dominant, we use satellite data from the Tropospheric Monitoring Instrument (TROPOMI) on board of the European Sentinel 5 Precursor (S5P) satellite (Veefkind et al., 2012), which was launched in 2017. The CH_4_ observations of TROPOMI have a spatial resolution of approximately 7 × 5.5 km^2^ in nadir and larger toward the edges of the 2,600 km wide swath. The main contribution of TROPOMI for CH_4_ emission monitoring is the continuous mapping capability, providing a large number of overpasses over any given region on Earth. The spatial resolution is not sufficient to detect small individual plumes in the Permian, where most emissions are below 2,000 kg hr^−1^ (Cusworth et al., 2022). Instead of detecting individual plumes, the aim of this work is to derive CH_4_ emissions on a spatial resolution of approximately 10 km. TROPOMI data have been used for quantifying emissions for the Permian basin using the wind rotation method (Schneising et al., 2020), Bayesian inversion involving chemistry‐transport modeling (Zhang et al., 2020) and the divergence method (Liu et al., 2021). The emission estimates of these studies are in the range of 2–4 Tg yr^−1^ CH_4_ for the period 2018–2019.

In this work, we use the divergence method with a new data‐driven approach to derive the large CH_4_ background column, which removes the need for model estimates of the background. Before applying it to satellite data, we verify the method using model data. The spatial distribution of the TROPOMI derived emissions is compared to oil and gas production and drilling information and with TROPOMI derived NO_x_ emissions. A time series analysis for locations in the Permian basin with large emissions is presented, as well as the estimate of the emissions of the entire Permian basin.

## 2 Materials and Methods

In this section, we describe the methods that are used to derive emissions from the column‐averaged dry air mole fraction of methane (XCH4), which involves two steps: first the CH_4_ background and the enhanced column densities are computed, next the divergence is applied to estimate the emissions.

### 2.1 Background Correction

The aim of the background correction is to convert the XCH4 volume mixing ratio into a background CH_4_ column density and a lower tropospheric enhancement. Therefore, we first convert the XCH4 volume mixing ratio into the CH_4_ column density, *n*, in units of mol m^−2^, by multiplying with the dry air number density:

(1) $$n=10^{-9}\;XCH4\;\frac{p_{d,sfc}}{g\;m_{air}}$$

where *n* is the CH_4_ column density in units of mol m^−2^, *p*_*d*,*sfc*_ is the dry surface pressure in Pa, *g* is gravitational constant, estimated as 9.81 m^2^ s^−1^, and *m*_air_ is the molar mass of dry air (28.96 10^−3^ kg mol^−1^).

**Figure 1:**
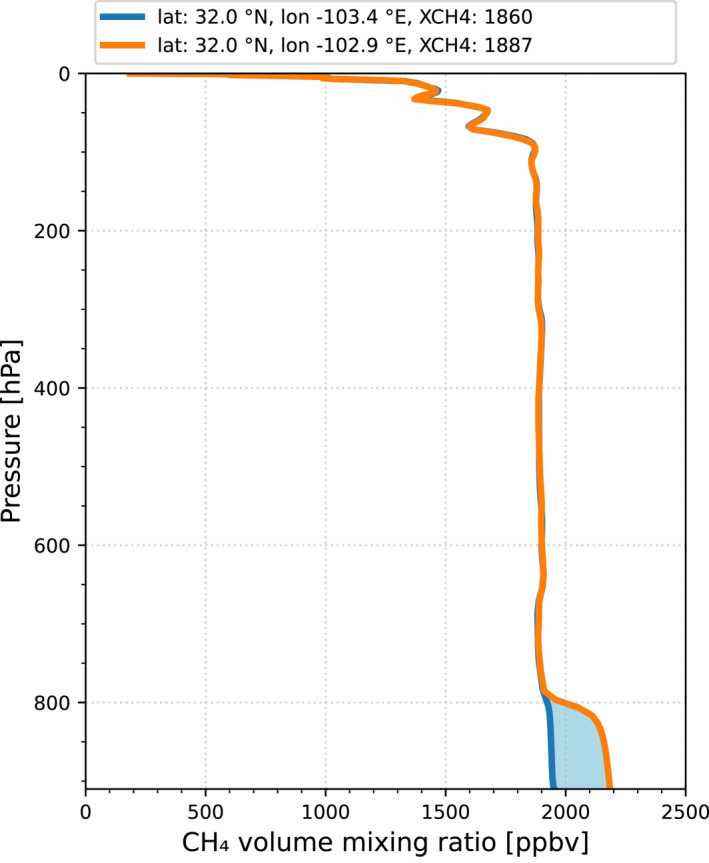
CAMS model (see Section 3) CH_4_ volume mixing ratio profiles for two nearby locations for 1 October 2020. The blue line represents a profile with background conditions and the orange line a profile with enhanced CH_4_ concentrations in the lower troposphere. The difference between the profiles is indicated by the light blue area.

Since this study focuses on a small spatial domain, the daily transport in the upper atmosphere, which was estimated by the daily model re‐analysis by Liu et al. (2021), can be simplified. To estimate the background CH_4_ column density, a simple model is applied that describes the CH_4_ column density as the sum of a stratospheric contribution, a background tropospheric contribution and a lower tropospheric enhancement. This model is illustrated in Figure 1, which shows model data for a profile with and without lower tropospheric enhancement from the CAMS (Copernicus Atmosphere Monitoring Service) global forecasting system (Agustí‐Panareda et al., 2019). The bulk of the CH_4_ column is determined by the tropospheric background, for which the concentration is almost constant from the surface to the tropopause (∼100 hPa for this profile). The concentration in the stratosphere decreases with altitude. For the example shown in Figure 1, the contribution of the enhancement for pressure ranges above 800 hPa contributes 1.4% to the column integrated XCH4.

Using the model described above, the CH_4_ column density can be written as:

(2) $$n=k\;XCH4_{s}\;p_{d,tp}+k\;{XCH4}_{t}\;\left(p_{d,sfc}-p_{d,tp}\right)+∆n$$

where $k=\frac{10^{-9}}{g\;m_{air}}$, XCH4_*s*_ is the mean CH_4_ dry air mole fraction in the stratosphere, XCH4_*t*_ is the mean CH_4_ dry air mole fraction in the troposphere, *p*_*d*,*tp*_ is the dry tropopause pressure and Δ*n* is the lower tropospheric CH_4_ enhancement.

For a limited‐sized area, we assume that the tropopause pressure, XCH4_*s*_ and XCH4_*t*_ are constant over the area, whereas Δ*n* is expected to vary. Although stratospheric intrusions are common in the western USA (e.g., Lin et al., 2012), especially in spring, the Permian basin is at the eastern end of the affected area and therefore the impact of the assumption of a constant tropopause pressure will hold on most days. Under these assumptions, Equation 2 can be rewritten as:

(3) $$∆n=n-\left(c_{0}+c_{1}\;p_{d,sfc}\right)$$

where $c_{0}=k\;\left(XCH4_{s}-{XCH4}_{t}\right)\;p_{d,tp}$ and $c_{1}=k\;XCH4_{t}$.

Thus, the background can be estimated by linearly fitting the CH_4_ column density *n* as a function of the surface pressure *p*_*d*,*sfc*_, yielding the constants *c*_0_ and *c*_1_. This is expected to provide an accurate estimate of the background CH_4_ column density when the lower tropospheric enhancement Δ*n* is close to zero for the larger part of the area. To make the fit less sensitive to the enhanced CH_4_ column density values, we first bin the data based on the surface pressure. For each bin, we compute the 25th percentile of the CH_4_ column density and the median of the dry surface pressure. These binned points are fitted using a linear least‐squares fit, yielding the parameters *c*_0_ and *c*_1_ of Equation 3, which describe the background CH_4_ column density.

**Figure 2:**
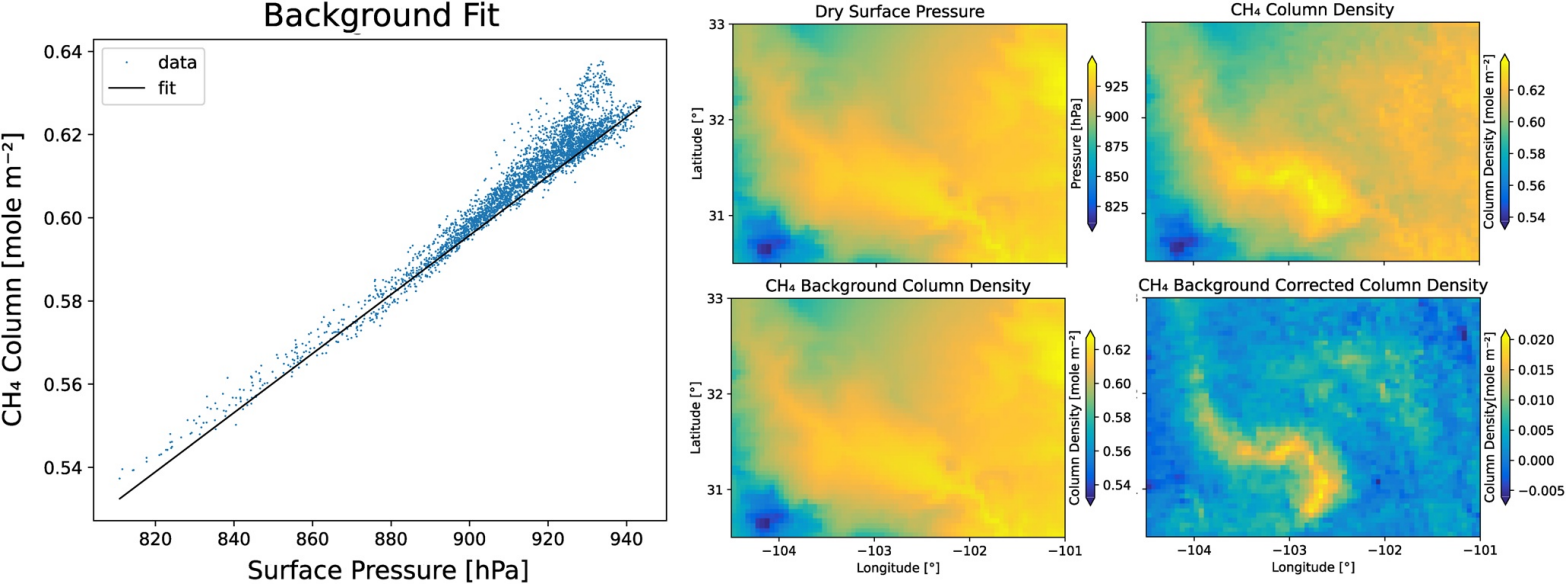
Illustration of the background correction for data over the Permian region for 6 October 2020. Left panel: CH_4_ column density plotted as a function of the surface pressure (blue points) and the corresponding linear fit (black line). Middle top panel: surface pressure. Right top panel: CH_4_ column density. Middle bottom panel: background CH_4_ column density. Right bottom: background corrected CH_4_ column density.

The method to estimate the background column density and the lower tropospheric enhancement is illustrated in Figure 2 for the Permian basin for 6 October 2020. In the figure, the CH_4_ column density is shown along with the linear fit represented by the line. The images show the constructed background column density and the background corrected CH_4_ column density (Δ*n*). It is noted that Δ*n* will show similar spatial features as XCH_4_, however it is now in number density units, which is required for the divergence method.

### 2.2 Divergence Method

Based on the conservation of mass, the emission can be computed as the horizontal flux divergence (Beirle et al., 2019), assuming that the sink term can be neglected due to the long atmospheric lifetime of CH_4_ (Liu et al., 2021). When considering a volume of air, we can compute the number of CH_4_ molecules that flow into and out of this volume from the column density and wind speed data. For steady state conditions, a positive difference indicates the presence of a CH_4_ emission source. From the continuity equation for steady state conditions, this emission *E* can be computed as:

(4) E≈∇⋅F=∇⋅(v∆n),

where F is the CH_4_ flux, ∆n is the aforementioned lower tropospheric enhancement of CH_4_, and *v* = (*u,v*) is the horizontal wind field. The horizontal divergence operator is defined as $\nabla =\left(\frac{\partial }{\partial x}\;i+\frac{\partial }{\partial y}\;j\right)$, where *x*, *y* are two perpendicular directions and **i** and **j** are unit vectors in the corresponding directions.

When applying Equation 4 for the lower tropospheric CH_4_ enhancements, the windspeed should be used for a representative altitude. As the bulk of the enhancement is expected in the boundary layer, we use as a baseline the average windspeed between the surface and 500 m altitude. Section 4 includes an analysis to test the sensitivity for the choice of the windspeed altitude. The divergence is computed using numerical derivatives calculated as the fourth‐order central‐finite difference.

The divergence method is extended here by introducing a second estimate of the divergence. The standard divergence method computes the divergence based on pixels in the S‐N and E‐W directions. While this method already has been proven very powerful, it does not make full use of the observations as it uses only half of the neighboring pixels. A second estimate of the divergence can be computed by using the ground pixels in the SW‐NE and NW‐SE directions (for a graphical representation see Figure S1 in Supporting Information S1). We compute the wind vector (*u’*,*v’*) on these axes, by applying a rotation of 45° with respect to the original wind vector (*u*,*v*). This provides two estimates of the divergence, which are combined by computing a weighted average. The weight depends on the wind direction and is computed for each grid box: when the wind is along the SN or EW directions, more weight is given to the original divergence, and when the wind is in the SW‐NE or NW‐SE directions more weight is given to the rotated divergence. The weight of the SN‐EW direction is given by:

(5) $$w_{0}=\left|\frac{\varphi }{45}-1\right|,$$

where φ is the angle in degrees between the vectors (u,v) and $\left(\bar{u},0\right)$. The weight for the NW‐SE direction is ${1-w}_{0.}$

Including the second estimate of the divergence has two advantages: first, it increases the data coverage because one of the divergence values may be missing due to missing or invalid input data, second it potentially reduces the noise, because we use twice as much data to compute the divergence.

### 2.3 Workflow

The following workflow has been developed to derive the CH_4_ emission in the Permian basin (see Figure S2 in Supporting Information S1 for a graphical representation). First, the daily XCH4 mixing ratios are background corrected yielding the tropospheric enhancements number densities. Filtering is applied to remove grid boxes for which the terrain height is varying strongly, as the background correction is expected to be inaccurate for these conditions. Additionally, this step avoids the posterior correction on the background correction, which is important in Liu et al. (2021). Next, the tropospheric enhancements are used by the divergence method to compute CH_4_ emissions for each day, providing the availability of sufficient cloud‐free observations. The emissions are filtered to only include grid boxes that have valid neighbors to remove effects of cloud edges, yielding for the Permian basin we typically emission estimates for 1/3rd of the days. Mean and median emissions are computed for the years 2019 and 2020 and for the entire period.

## 3 Data

S5P TROPOMI CH_4_ mixing ratios are from the Weighting Function Modified Differential Optical Absorption Spectroscopy (WFM‐DOAS) algorithm version 1.5 (Schneising et al., 2019). The main data fields used are the methane column‐averaged dry air mole fraction (XCH4), the surface pressure, the geolocation, and the data quality information. The WFM‐DOAS data were downloaded from https://www.iup.uni-bremen.de/carbon_ghg/products/tropomi_wfmd for the years 2019 and 2020.

From the global data, a daily gridded data set for the years 2019 and 2020 is derived on an equirectangular grid with a resolution of 0.05° in both latitude and longitude, which corresponds to 5.5 km in the N‐S direction and for a latitude of 30° to 4.8 km in the E‐W direction. Note that the resolution is similar to the nadir resolution of the TROPOMI CH_4_ observations (5.5 km in flight direction and 7 km in the cross‐flight direction). The gridding processing uses the ground pixel corners provided to calculate the overlap with the grid boxes, which are used to compute weighted averages. The gridding yields daily fields of XCH4 and surface pressure. Only ground pixels with the recommended data quality are included in the gridded fields.

Meridional and zonal wind components are from the ERA‐5 reanalysis from the European Centre for Medium‐Range Weather Forecasts (Hersbach et al., 2020). The wind data is downloaded for all 14 pressure levels between 600 and 1,000 hPa and has a spatial resolution of 0.25° × 0.25° latitude‐longitude and an hourly temporal resolution. In the divergence method, we take the wind history into account by averaging the wind data for the time steps of 17, 18, and 19 hr UTC.

Model XCH4 and surface pressure data have been used from the Copernicus Atmosphere Monitoring Service (CAMS) global forecasting system (IFS cycle 47R1) (Agustí‐Panareda et al., 2019), experiment he9h (Barré et al., 2021). Two satellite observation streams have been assimilated in this data set: the Infrared Atmospheric Sounding Interferometer (IASI) on the MetOp satellites and the Thermal and Near‐infrared Sensor for carbon Observations (TANSO) on the GOSAT satellite. In this data set, only the concentrations are adjusted by the assimilation; the emissions and surface fluxes remain unchanged. Due to their respective vertical sensitivities, the satellite data mainly provide a correction to the concentrations in the free troposphere and above, whereas at lower altitudes the emissions are the dominant influence on CH_4_ concentration. The CAMS data have a spatial resolution of 0.1° × 0.1° latitude × longitude. Data for each day of the year 2020 at 18:00 UTC were obtained and resampled to the same spatial grid as the TROPOMI XCH4 data. Also, all grid boxes for which the TROPOMI data has fill values were removed in the CAMS XCH4 data set, to generate representative pseudo‐observations. In addition to the CAMS XCH4 data, the CAMS emissions version 4.2 with a spatial resolution of 0.1° × 0.1° latitude × longitude has been used (Granier et al., 2019). The anthropogenic emissions in this data set, including fossil fuel, agricultural and landfill/waste emissions, are from EDGARv4.2FT2010 (Olivier & Janssens‐Maenhout, 2012). From the original monthly data, average emissions for the sum of all sectors for 2019 and 2020 have been constructed.

Oil and gas production data and drill rig counts are from the Enverus Drilling Info and Rig Analytics database tools (https://www.enverus.com/drillinginfo-and-rigdata/, last accessed 31‐10‐2022). Oil and gas production volumes are reported monthly for each well location and gridded to match the TROPOMI CH_4_ maps. The locations of drill rigs are reported daily. Monthly gridded maps are created by counting the number of drill rigs within each grid cell weighted by the number of days on location per month.

The NO_x_ emission data are from (Dix et al., 2022). These data represent a mean NO_x_ emission for the time period May 2018 until December 2020 and have been gridded to the same latitude‐longitude grid as the TROPOMI XCH4 data.

## 4 Verification of the Method Using CAMS Model Data

**Figure 3:**
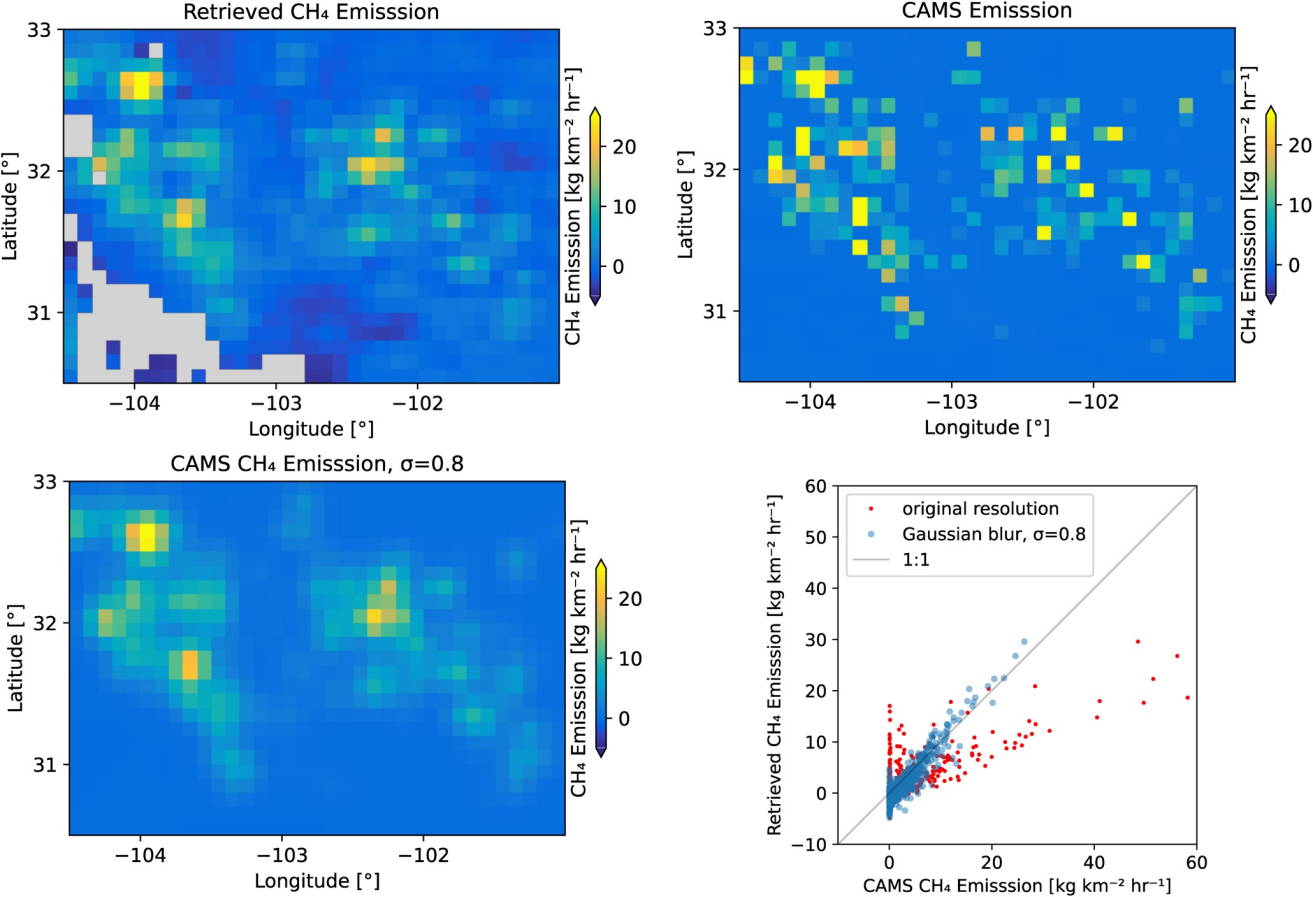
Divergence method applied to CAMS model data. Top‐left panel: CH_4_ emission derived from CAMS model data with the divergence method. Top‐right panel: CAMS input emissions on the original resolution. Bottom‐left panel: CAMS input emission with a Gaussian blur with *σ* = 0.8. Bottom‐right panel: retrieved emissions plotted as a function of the CAMS input emissions, for the original and blurred data.

To verify that the divergence method can be used to estimate the CH_4_ emissions, we first applied it to CAMS model data, for which the emissions are known. This verification is limited to provide credibility for the methodology, as we know that the CAMS emissions for the Permian basin are not up‐to‐date. As described above, the CAMS data for 2020 were gridded and sampled for the grid boxes for which also TROPOMI XCH_4_ is available. Thus, it has the same coverage as the TROPOMI, including missing data due to, for example, cloud contamination. On this data set we apply the same background correction and derive the emissions using the divergence method. Figure 3 shows the median emission for 2020 derived from the CAMS data as well as the input emissions. The retrieved emissions show generally the same spatial features as the input emissions, however at a lower spatial resolution. To test this, we applied a Gaussian blur to the input emissions. We manually varied the standard deviation σ of the Gaussian kernel and found that for a value of approximately 9 km the resolution of the retrieved and input emissions match well. The spatial resolution has a strong effect on the slope between the retrieved and input emissions, and the correlation coefficient increases from 0.75 to 0.93 when the Gaussian blur is applied. Whereas the divergence method favorably retrieves the spatial variability of the emissions, retrieving the total emission is more complicated. We computed the total emission for the Delaware and Midland sub‐basins, as well as for the entire Permian basin (the definition of these regions is shown in Figure 5). The estimated emission for the Delaware sub‐basin shows a bias of −16% compared to the CAMS inputs, for the Midland sub‐basin −40% and for the entire Permian basin −42%. The total emission estimates are sensitive to a possible offset: applying an offset of 1.0 kg km^−2^ hr^−1^ to the retrieved emissions is sufficient to close the gap between the retrieval and the input. The bias with this offset applied is 7%, −8%, and −1%, for the Delaware sub‐basin, the Midland sub‐basin and the entire Permian basin. It is noted that the mean emission of the Delaware sub‐basin is almost a factor of 2 larger compared to the mean for the entire Permian basin and for the Midland sub‐basin this is a factor of 2.6. For larger emissions, a bias will have a smaller relative impact than for lower emissions, which agrees with the finding that the bias is lower for the Delaware sub‐basin. Thus, these results point to a possible low bias of the method, which is a potential limitation of the method for estimating the total emission of a region, especially when the average emissions are small. Small emissions over a large part of the region of interest will result in a constant background concentration, which may be removed by the background correction, leading to a low bias of the emissions. In addition, a possible complication of this analysis is that in the CAMS data set the concentrations and emissions are not consistent, because only the concentrations are adjusted using satellite observations. However, it is not straightforward to assess the sign or magnitude that this may have on the emissions derived with the divergence method.

**Figure 4:**
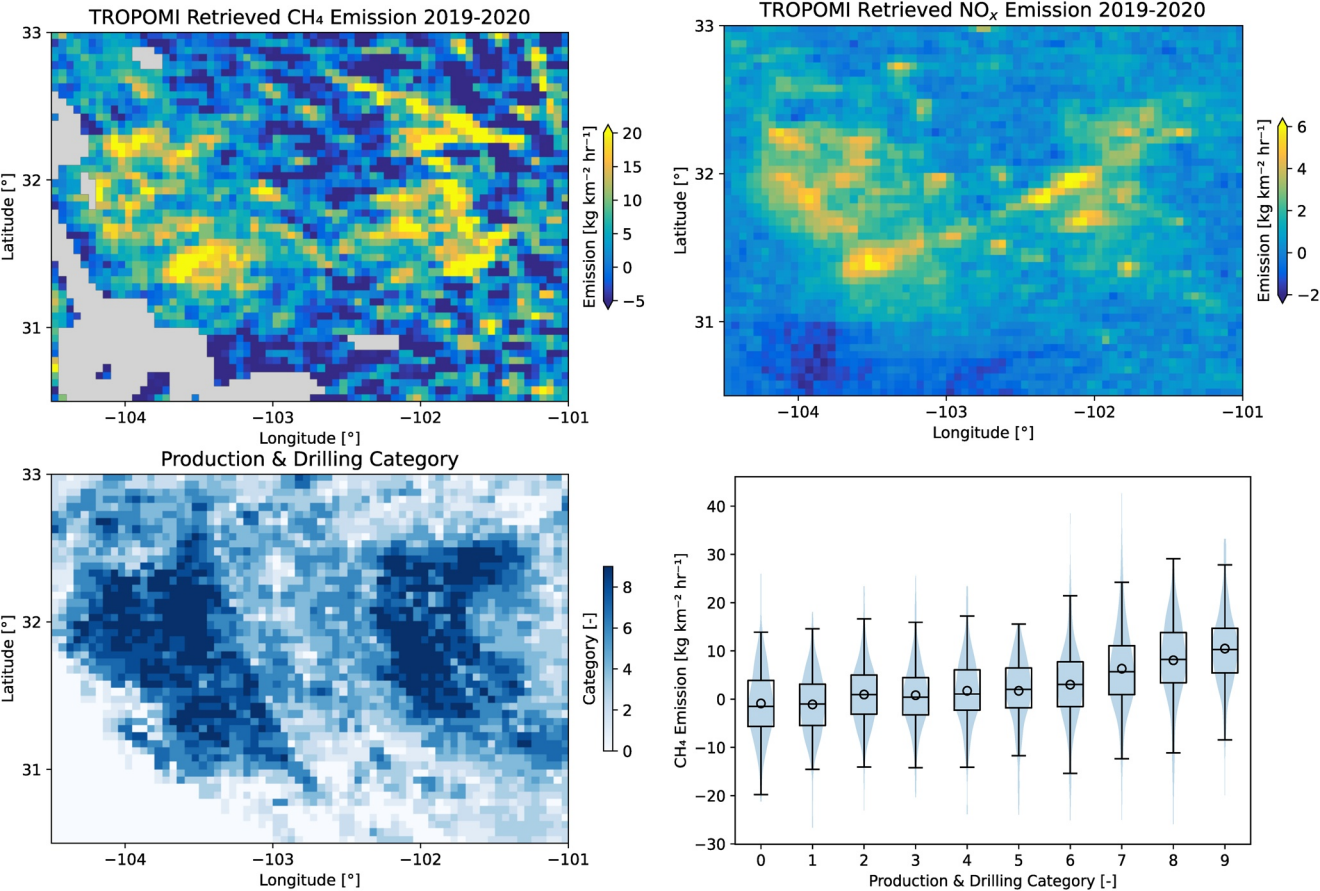
Panel top left: Median CH_4_ emission derived using the divergence method applied to TROPOMI data from 2019 to 2020. Top‐right NO_x_ emission for 2018–2020 retrieved from TROPOMI (Dix et al., 2022). Bottom left: production drilling categories. Bottom right, combined violin and boxplot showing the distribution of retrieved CH_4_ emissions for each production/drilling category.

**Figure 5:**
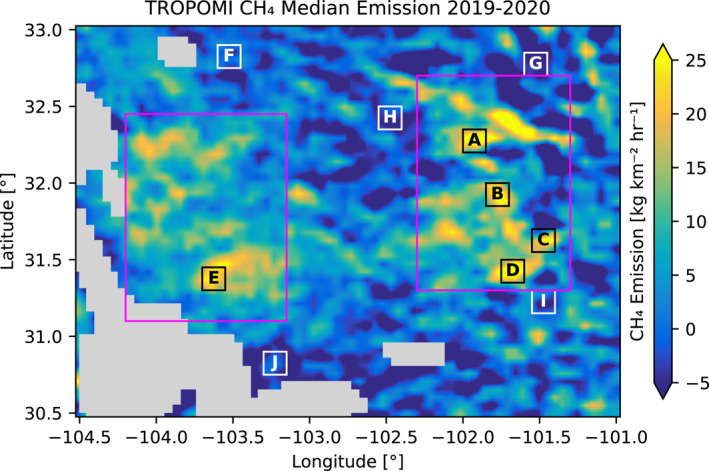
CH_4_ median emission with the five selected locations with high emissions (labeled A–E in black) and background emissions (labeled F–J in white). The pink boxes indicate the areas used for computing the statistics for the Delaware and Midland sub‐basins.

The CAMS data have also been used to test the sensitivity for variations in the setup. The default divergence setup uses a boundary layer height of 500 m and a fourth‐order central‐finite difference method for calculating the derivatives. We have tested the impact of boundary layer heights of 250 and 1,000 m, the use of second‐order central‐finite difference, and the use of a least‐squares fit (instead of the fit based on the 25th percentile) to compute the background. The boundary layer height is important because the wind is computed as the mean over this layer. As can be seen in Table 1, the largest impact on the mean emission is due to the background method (−19% compared to the default) and increasing the boundary layer to 1,000 m (−11%). The impact of changing the boundary layer to 250 m and the impact of the central‐finite difference method order is marginal (<2%). These results are similar to the results presented by Liu et al. (2021). Based on this sensitivity analysis, we estimate the uncertainty of the mean emissions of the order 25%.

**Table 1:** Sensitivity of the Emissions for 2020 Derived From the CAMS Data for the Entire Domain, for Variations in the Assumed Boundary Layer Height, the Order of the Central‐Finite Difference Method and the Method to Compute the Background

Emission (kg m^−2^ hr^−1^)	Default	250 m	1,000 m	Second order	Lstsq
Mean	1.44	1.47	1.28	1.43	1.17
Median	0.16	0.23	0.01	0.21	−0.20
P1	−0.90	−0.84	−1.04	−0.85	−1.08
P3	2.20	2.19	2.10	2.24	1.90

*Note*. The first column represents the default case, which uses a boundary layer height of 500 m, fourth order central finite difference and a fit of the background column density based on the 25th percentiles. Variations with respect to the default are a 250 m or 1,000 m boundary layer height, a second order central‐finite difference method, and a least‐squares fit (Lstsq) for the background column density. The table lists the mean, median, 25th percentile (P1) and 75th percentile (P3) of the CH_4_ emission over the Permian basin.

## 5 CH_4_ Emissions Derived From TROPOMI Data

In this section, spatially resolved CH_4_ emission maps over the Permian basin are presented and compared to NO_x_ emissions, industry activity data and other top‐down emission estimates. The median CH_4_ emissions derived by applying the divergence method to TROPOMI WFMD data for 2019–2020 are shown in Figure 4. Overall, the emission is highest in regions where there are activities related to the O&G industry. The spatial outline of the Delaware and Midland sub‐basins can be clearly distinguished in this figure. Whereas the main outlines correspond to the emission data derived from the CAMS model data (Figure 3, top left panel), there are significant differences between these maps. The TROPOMI‐derived emissions show significantly higher spatial variability compared to the emissions derived from the CAMS model data. This may be caused by the instrument noise, which is not accounted for in the CAMS analysis. In addition, the CAMS model assumes a fixed pattern, whereas in reality, the emissions will vary significantly in space and time. Also, for the CAMS data the wind information and the advection in the model are consistent, whereas for the application on TROPOMI data there may be significant errors in the wind fields. Some regions in Figure 4 show negative emissions, which are considered artifacts of the method. Analyses indicate that strong variations in orography and surface albedo could lead to significant negative emissions, but other unknown effects could also play a role. For regions with orography, the implicit assumption that the lower tropospheric enhancements will follow the terrain variations may not hold. In reality, when the orography is strong, (part of) the enhancements will be trapped in the valleys, resulting in spatial variabilities in the CH_4_ column density. The divergence method will interpret these as negative and positive emissions. The link between negative emissions and surface albedo variations is less well understood. Albedo dependent biases of the satellite product are addressed by a machine learning calibration and are expected to be marginal in the Permian. Other more complex effects could be a correlation between orography and surface albedo effects, for example, bare rock or snow on mountain tops versus vegetation in the valleys, or a link between surface albedo and surface roughness affecting the atmospheric transport.

The NO_x_ emissions in the Permian basin are to a large extent related to the O&G industry. The NO_x_ and CH_4_ emissions derived from TROPOMI (Figure 4) show similar large scale spatial structures and moderate spatial correlation with correlation coefficients of 0.59, 0.38 and 0.47, for the Delaware sub‐basin, the Midland sub‐basin and the entire Permian basin. The spatial correlation is higher in the western Delaware sub‐basin compared to the Midland sub‐basin. In the Midland sub‐basin, there are significant NO_x_ emissions from road transportation and power generation related to the main cities, Midland and Odessa and the Interstate I‐20 (Dix et al., 2022). As expected these features are not found in the CH_4_ emission data.

To further link the satellite derived CH_4_ emission data with the O&G industry activities, we have used data on the oil and gas production and on the drilling days. The relative contributions of oil and gas production and drilling to CH_4_ emissions are not known and may vary between facilities and over time, which prevents a quantitative analysis. Instead, we applied semi‐quantitative comparison, where we categorized the activity data in the following way. For each grid box we define a score of 0–3 for oil production, gas production and drilling. A score of 0 is given when the activity data are less than 1% of the median, a score of 1 when the data are higher than this value but less than the 25th percentile, a score of 2 when the data are between the 25th and 75th percentile, and a score 3 when the data are higher than the 75th percentile. Finally, the scores of oil production, gas production and drilling are combined, which gives 10 categories ranging from 0 to 9. The map of the categories and the distribution of the CH_4_ emissions over the categories are shown in Figure 4. As can be seen in the figure, the overall spatial variation of the production and drilling data shows good agreement with both the CH_4_ and NO_x_ emissions. Especially for categories 7–9 significantly higher CH_4_ and NO_x_ emissions are found and the lower categories show an average value of near zero, which is also a sign that the satellite retrievals are in correspondence with the oil and gas activity data.

**Figure 6:**
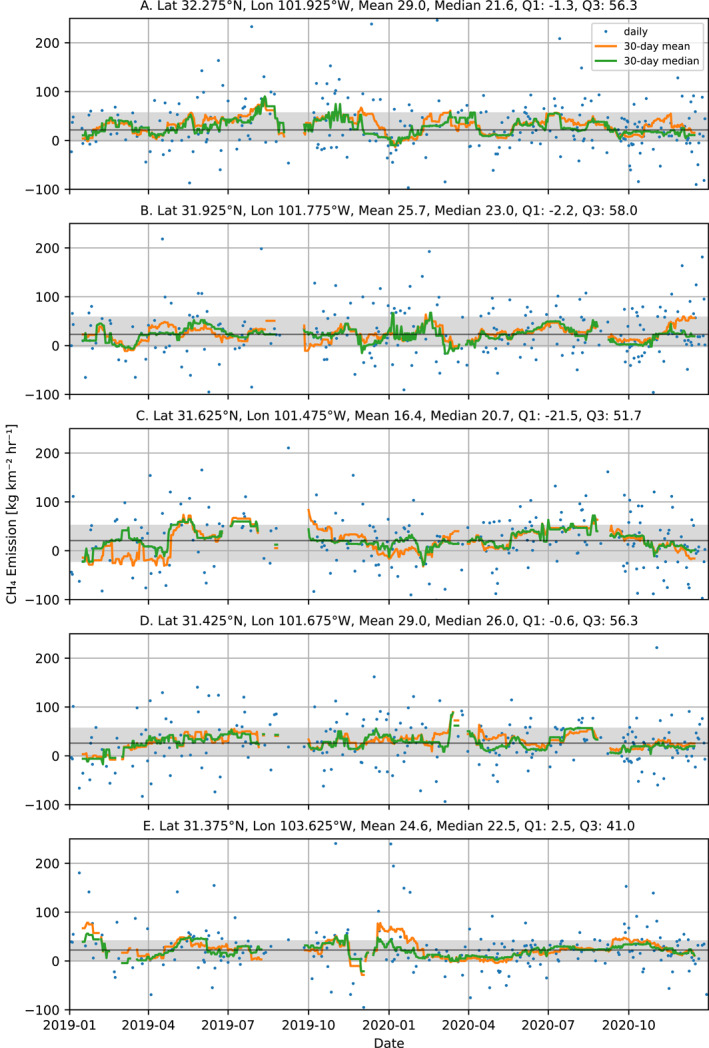
Time series for five locations with high CH_4_ emissions. The blue dots are the daily data, the orange line the 30‐day running mean and the green line represents the 30‐day running median. Running mean and median are only shown when at least 5 of the 30 days contain valid data. The gray area indicates the interquartile range and the black line is the median over the whole time period.

For five locations with high median emissions selected by hand (labeled A–E in Figure 5) time series of the CH_4_ emission for 2019 and 2020 are shown in Figure 6. For all locations, except for location C, the 30‐day running mean and 30‐day running median are above zero for almost the entire time period. As can be seen in Figure 5, there is an area with negative median emissions just north of the location C. These negative emissions are probably an artifact of the orography and are also affect the time series for the location C. For the other four locations in Figure 6, the mean emission over the whole time period is larger than the median value, indicating that the distribution is skewed toward the larger values. Although the mean value is much more sensitive to outliers compared to the median, the mean falls well in the interquartile range. Furthermore, the difference between the mean and median is less than 34% for the five locations. The median value is more representative for the continuous emissions and less sensitive to extreme values compared to the mean. For reference, we analyzed time series for background conditions (locations F‐J in Figure 5). For these background locations, the difference between the mean and median over the entire time period is smaller as compared to the locations with high emissions (see Figure S3 in Supporting Information S10).

We also analyzed the distribution of the daily emissions for the Permian basin and the sub‐basins. Overall, the distributions are heavy tailed and skewed toward the large values. For the 2019–2020 period the mean is 30% higher than the median for the entire Permian and 24% and 27% for Delaware and Midland sub‐basins, respectively.

Super emitting events that are short in duration will have a much larger effect on the mean than on the median and will result in a different skewness of the distribution. We found that the mean and median differ by less than 34% for both the time series for high emitting locations as well as for the entire Permian basin. To further analyze these results, we simulated time series, where we varied the percentage of days on which emissions occur from 2% to 100% of the days, while ensuring that the mean emission over the time series is the same for all cases. Noise was added using either a bootstrap method using the background data from locations F–J in Figure 5, or randomly with a standard deviation of 25 kg km^−2^ hr^−1^. Each case was repeated 1,000 times to collect sufficient statistics. This analysis indicated that the difference that we report between the mean and median is explained for the cases when we add the emission on at least 35% of the 250 days. When looking at the skewness of the distributions we found that the values in the enhanced regions match best with emissions on at least 10% of the days. This indicates that the emissions are frequent, which is further supported by comparing monthly maps, which show similar spatial patterns for the main CH_4_ emission hotspots.

From the daily emissions, we estimated the annual emissions for three regions: the Delaware sub‐basin, the Midland sub‐basin and the entire Permian. The boundaries used for the Delaware and Midland sub‐basins are shown in Figure 5, the Permian basin covers the entire map. The annual emissions, listed in Table 2 for 2019, 2020, and 2019–2020, were calculated by summing the yearly mean values of the basins and converting them to Tg yr^−1^. Based on the sensitivity analysis presented in Section 2, we estimate the uncertainty in these numbers as 25%. This uncertainty is dominated by biases caused by the configuration of the method, predominantly the selected altitude range for which the mean wind is computed, and the method for computing the background CH_4_ column. Therefore, the uncertainties in the annual emissions are expected to be significantly larger compared to differences in the annual emissions between the years. The estimated emission for the Permian basin agrees within the uncertainties with estimates from Liu et al. (2021) (2.82–3.78 Tg yr^−1^), (Schneising et al., 2020) (3.18 ± 1.13 Tg yr^−1^ for 2018–2019) and (Zhang et al., 2020) (2.9 ± 0.5 Tg yr^−1^ for March 2018–2019). The annual CH_4_ emissions of the Delaware and the Midland basins are found to be comparable, and it is estimated that these sub‐basins contribute 70%–90% to the entire CH_4_ emissions of the Permian basin. The derived emissions are found to be 9%–27% lower in 2020 compared to 2019, where the decrease is larger for the sub‐basins compared to the entire Permian basin. This decrease is in agreement with results from the wind‐rotation method of Schneising et al. (2020), which shows a decrease of 22% for the Permian basin. These decreases may be an indication of the impact of the COVID‐19 crisis (Lyon et al., 2021), however effects of interannual variability and effects of sampling and noise could also explain such variations.

**Table 2:** Annual Emissions Derived With the Divergence Method for the Delaware, Midland Sub‐Basins and for the Entire Permian Basin for 2019, 2020 and 2019–2020

Emission (Tg yr^−1^)	Delaware^a^	Midland^a^	Permian		
			This work^a^	Wind rotation^b^	Divergence^c^
2019	1.4	1.2	3.0	2.9 ± 1.6	3.1 (2.8–3.8)
2020	1.1	0.9	2.8	2.3 ± 1.7	–
2019–2020	1.3	1.0	2.9	–	–
Difference 2020–2019	−19%	−27%	−9%	−22%	–

*Note.* The Delaware and Midland basins are sub‐basins of the Permian (see Figure 5). Also included are results using the wind rotation technique (using the method described in Schneising et al. (2020)) and results from Liu et al. (2021) using an alternative implementation of the divergence method.

^a^ Estimated 1‐σ uncertainty of the emission is 25%.

^b^ Updated results using the method of Schneising et al. (2020).

^c^ (Liu et al., 2021).

## 6 Conclusions

Global monitoring of CH_4_ emissions is an essential element of climate change mitigation strategies. We have developed a method to derive spatially resolved CH_4_ emissions using satellite data from TROPOMI on the Sentinel 5 Precursor satellite, which we verified and applied for the Permian basin in the USA for the years 2019–2020. Compared to previous applications of the divergence method for CH_4_ emissions (Liu et al., 2021), we use a model‐independent correction to derive the CH_4_ background column density based on assuming that the background CH_4_ concertation in the troposphere and the tropopause height is spatially constant and is therefore suited for regional applications. Using model data for 2020, we demonstrated that the divergence method can retrieve the spatial variability of the emissions at a reduced spatial resolution of approximately 10 × 10 km^2^. Based on sensitivity analyses, we estimate that the uncertainty in the yearly mean emissions is of the order of 25%.

For implementing CH_4_ mitigation strategies, it is important if emissions are caused by a few very large infrequent events, few frequent emissions, or by widespread frequent emissions. Although the spatial resolution of the TROPOMI does not allow the observation of smaller individual plumes, we find emissions at a 10 × 10 km^2^ scale throughout the entire Permian basin. Analysis of time series for large emission locations indicates that these emissions occur for at least 10%–35% of the days. These results are in line with the scenario of widespread frequent emissions and also in agreement with (Schneising et al., 2020) for the entire Permian basin. However, we cannot conclude if a scenario of thousands of small emissions or a limited number of larger leaks is dominating the CH_4_ emissions.

The retrieved CH_4_ emission maps show moderate spatial correlation with NO_x_ emissions derived from TROPOMI and with information on oil and gas production and drilling activities. Therefore, constructing CH_4_ emissions from these data sets, as suggested by De Gouw et al. (2020), will require additional information, for example, locally varying conversion factors. Also, deriving direct relationships between oil and gas production and emissions is not straightforward, as the emission rate may vary between the thousands of production sites in the Permian basin. Furthermore, it was found that the spatial variability of the retrieved emissions differs significantly from the emission inventory used by CAMS (Granier et al., 2019).

The derived annual emissions show agreement with other satellite‐derived emissions for the Permian basin. The spatially resolved results allow to estimate the annual emissions of the Delaware and Midland sub‐basins separately and showed to be of similar magnitude. Annual emissions in the Permian basin for the year 2020 are 9%–27% lower compared to 2019, which may be a result of the drop in demand for oil and gas due to the COVID‐19 crisis (Lyon et al., 2021), however, it is not clear if these reductions are robust given the noise, sparse sampling, and uncertainty in the applied method.

In this work, we applied the divergence method to the Permian basin, which is an important and well‐studied region for CH_4_ emissions. The presented method is generally applicable and may also be used in other oil and gas production regions as well as other satellite sensors and can be implemented to provide up‐to‐date information on the success of CH_4_ mitigation policies. The divergence method is based on spatial variability of the CH_4_ concentration observations. Therefore, the method is expected to perform best in regions with strong spatial variability in CH_4_ emissions, limited spatial variability and sufficient cloud‐free observations.

## Supporting information

Supporting Information S1

## Data Availability

The main data sets that are used in this research are: The TROPOMI WFM‐DOAS (University of Bremen IUP, 2021). The ERA‐5 meteorological information (ECMWF, 2021). The CAMS model data (CAMS, 2021). All processed data sets created as part of this work, as well scripts for data analysis and the generation of the figures, are available as a publication replication package (Veefkind, 2023).
