# Supplementary material for: Widespread Frequent Methane Emissions From the Oil and Gas Industry in the Permian Basin
Source: J Geophys Res Atmos. 2023 Jan 31;128(3):e2022JD037479. doi: 10.1029/2022JD037479 (PMC10078246; doi:10.1029/2022JD037479)
Supplement: Supplementary file 1 — Supporting Information S1 [file JGRD-128-0-s001.pdf]

AGU PUBLICATIONS

JGR Atmospheres

Supporting Information for

Widespread Frequent Methane Emissions from the Oil and Gas Industry in the Permian Basin

J. P. Veefkind[1,2], R. Serrano-Calvo[2], J. de Gouw[3,4], B. Dix[3], O. Schneising[5], M. Buchwitz[5], J. Barré[6], R.J. van der A[1], M. Liu[1], P.F. Levelt[1,2,7]

[1] Royal Netherlands Meteorological Institute KNMI, De Bilt, The Netherlands.

[2] Delft University of Technology, Dept. Geoscience and Remote Sensing, Delft, The Netherlands.

[3] Cooperative Institute for Research in Environmental Sciences, University of Colorado, Boulder, CO, United States.

[4] Department of Chemistry, University of Colorado, Boulder, CO, United States.

[5] Institute of Environmental Physics (IUP), University of Bremen FB1, Bremen, Germany.

[6] University Cooperation for Atmospheric Research, Boulder, CO, United States.

[7] National Center for Atmospheric Research, Boulder, CO, United States.

Contents of this file

Figures S1 to S3

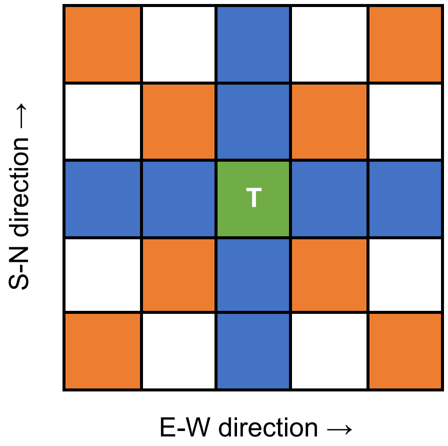

Figure S1. Schematic view of the ground pixels used to compute the divergence. For the green target pixel the standard divergence is computed based on the blue pixels. The rotated divergence is computed based on the orange pixels.

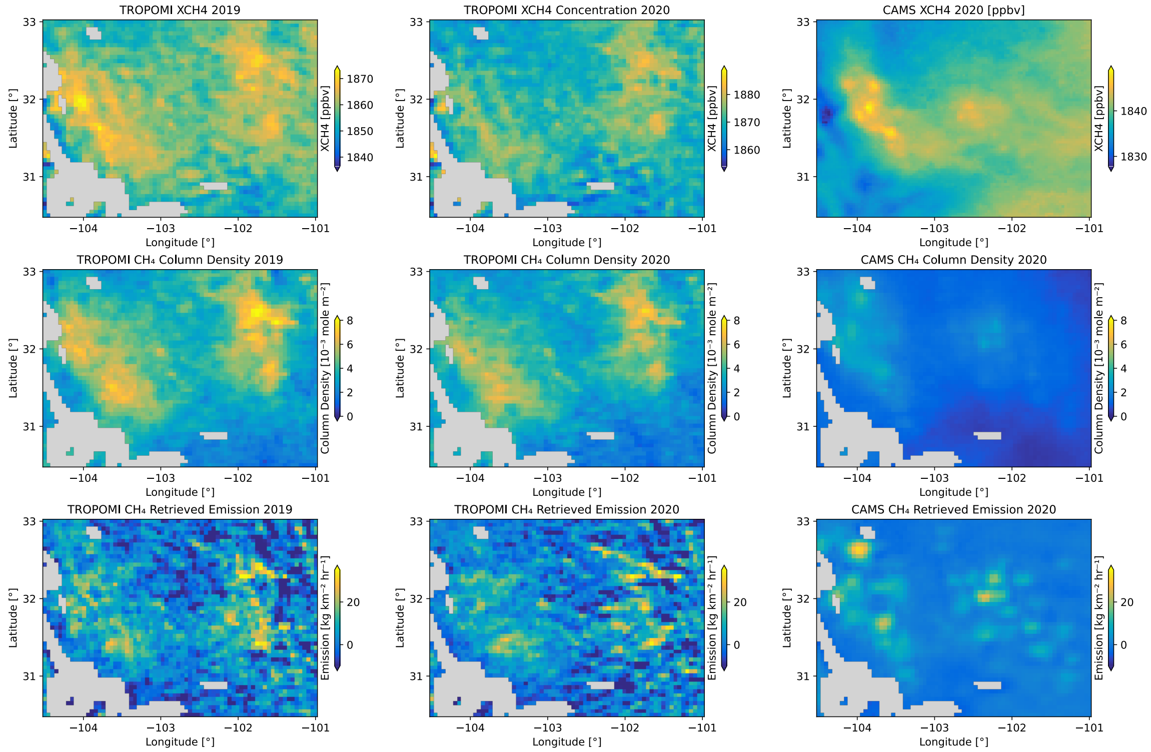

Figure S2. Top row: median XCH4 for Tropomi for 2019 (left), 2020 (middle) and for the CAMS model data for 2020. Middle row: same as top row, but for the background corrected CH_4 column concentrations. Bottom row: same as top row, but for the CH4 emissions derived with the divergence method.

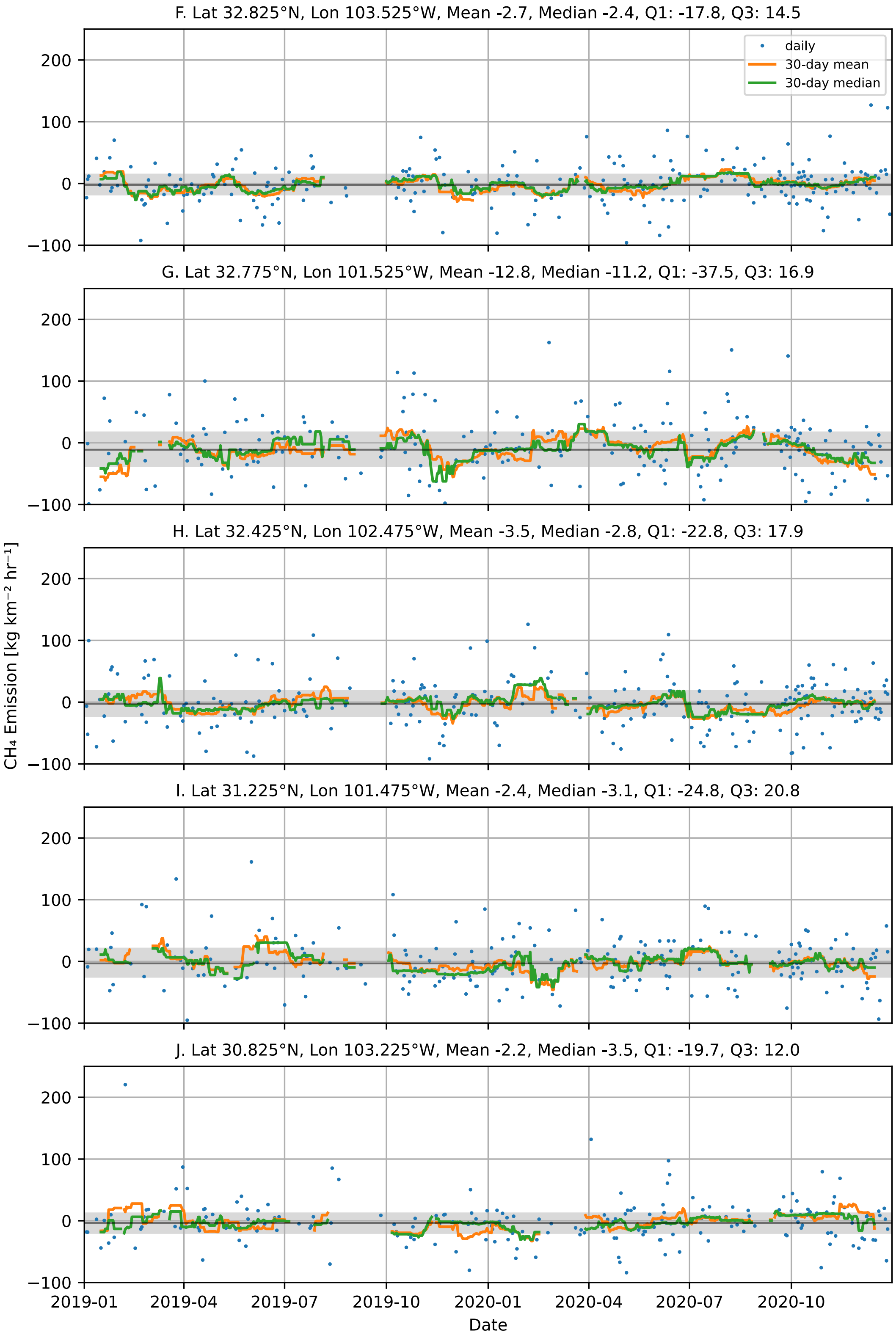
F. Lat 32.825°N, Lon 103.525°W, Mean -2.7, Median -2.4, Q1: -17.8, Q3: 14.5
daily
30-day mean
30-day median
G. Lat 32.775°N, Lon 101.525°W, Mean -12.8, Median -11.2, Q1: -37.5, Q3: 16.9
H. Lat 32.425°N, Lon 102.475°W, Mean -3.5, Median -2.8, Q1: -22.8, Q3: 17.9
I. Lat 31.225°N, Lon 101.475°W, Mean -2.4, Median -3.1, Q1: -24.8, Q3: 20.8
J. Lat 30.825°N, Lon 103.225°W, Mean -2.2, Median -3.5, Q1: -19.7, Q3: 12.0
CH₄ Emission [kg km⁻² hr⁻¹]
200
100
0
−100
2019-01
2019-04
2019-07
2019-10
2020-01
2020-04
2020-07
2020-10
Date

Figure S3. Time series for five locations with background CH_4 emissions. The blue dots are the daily data, the orange line the 30-day running mean and the green line represents the 30-day running median. Running mean and medians are only shown when at least 5 of the 30 days contain valid data. The grey area indicates the interquartile range and the black line the median over the whole time period.
